# Supplementary material for: Comprehensive characterization of the effect of mineralocorticoid receptor antagonism with spironolactone on the renin-angiotensin-aldosterone system in healthy dogs
Source: PLoS One. 2024 Feb 23;19(2):e0298030. doi: 10.1371/journal.pone.0298030 (PMC10890738; doi:10.1371/journal.pone.0298030)
Supplement: Attachment — Submitted filename: Response to Reviewers.pdf [file pone.0298030.s002.pdf]

Manuscript: Comprehensive characterization of the effect of mineralocorticoid receptor antagonism with spironolactone on the renin-angiotensin-aldosterone system in healthy dogs.

Response to PLOS ONE reviewers: PONE-D-23-07873

Dear PLOS ONE reviewers,

Thank you for the constructive reviews and edits to our manuscript "Comprehensive characterization of the effect of mineralocorticoid receptor antagonism with spironolactone on the renin-angiotensin-aldosterone system in healthy dogs." We appreciate the time and effort that you put into reviewing our manuscript and providing insightful feedback. Please see our responses below, in bold, to the comments provided.

Reviewer #1:

1. This is an interesΘng study, using sophisΘcated techniques and analyΘcal methods not ordinarily available to most veterinary research laboratories. Therefore, this study is valuable to report findings on the effects that ordinarily would not have been possible. However, the high degree of variability and lack of significance in many of the parameters measured led to findings and conclusions that are somewhat underwhelming. Perhaps the most significant limitaΘon of this study is that the invesΘgators used healthy Beagle dogs for their analysis. (1) Beagle dogs are known to respond to drugs differently and have different metabolism than other dogs. (2) healthy dogs likely respond to spironolactone (and other cardiovascular drugs) differently than dogs with heart disease. I fully agree with the authors (line 353) that "…the results of our study suggest that in healthy dogs without background RAAS acΘvaΘon [and heart disease] the physiologic effects of spironolactone are modest". You should include "insignificant for most measures in this study".

The modifier "and insignificant for most measures in this study" has been added to the statement previously on line 353.

2. Line 166: PharmacokineΘc analysis. You did not describe to the readers why you measured these spironolactone metabolites and did not measure spironolactone. The readers need more informaΘon. Is this because spironolactone is rapidly metabolized and not detected as the parent drug? Did you look for spironolactone in plasma of treated dogs and didn't find it? Because you already have a LCMS assay developed, it would have been easy to include detecΘon of spironolactone in your procedure. Explain why this was not done.

You are correct, spironolactone is a prodrug with a short plasma half-life (less than two hours). It rapidly undergoes hepatic metabolism, resulting in the formation of several primary metabolites, two of which act as a major active metabolites: 7α-thiomethyl-spironolactone (TMS) and the prominent dethioacetylated metabolite, canrenone. These active metabolites have a half-life estimated at around 15 – 20 hours in humans. Clarification regarding these metabolites was added to the manuscript under "Pharmacokinetic Analysis."

References have been added to the manuscript and appear below:
Kolkhof P, Bärfacker L. 30 years of the mineralocorticoid receptor: mineralocorticoid receptor antagonists: 60 years of research and development. J Endocrinol. 2017 Jul;234(1):T125-T140. doi: 10.1530/JOE-16-0600. PMCID: 28634268. PMID: PMC5488394

Struthers AD, Unger T. Physiology of aldosterone and pharmacology of aldosterone blockers. Eur Heart J Supplements, Volume 13, Issue supple B, July 2011, Pages B27-B30, https://doi.org/10.1093/eurheartj/sur009.

3. Line 172: In this secΘon the analyΘcal methods are described. The assay appears to be very complete and adequately validated. However, the reader of the paper may need more informaΘon if they were to duplicate this assay. It says "Chromatographic separaΘon was achieved isocraΘcally on a C18 column 2.1x50 mmn, 1.7 µm at 0.40 mL/min. The mobile phase contained water, acetonitrile and formic acid." Please list the source of column you used and specific packing. List the proporΘon (percent) of each component of your mobile phase. Likewise, in the next secΘon you didn't list the ions you monitored (m/z) ions or ranges. Please also list the lower limit of quanΘficaΘon for your assay. You used a signal/noise raΘo (s/n) of 5 for the LOQ. Is this standard for your lab? Seems a bit low for some guidelines.
Additional information was provided to allow for accurate duplication of the pharmacokinetic analysis performed in this study. Specifically, the source of the column (Acquity UPLC), proportion of each component of the mobile phase (70/30/0.1), and the ions monitored (canrenone 341>107, TMS 389>341, and canrenone-d6 347>107) were added to the revised manuscript. The lower limit of detection was 2 ng/mL. The signal/noise ratio statement was removed as the specificity of the method is more appropriately described in the sentence prior which states "the intra-assay precisions, based on three levels of QC samples (low, medium and high), were within 4.62 % CV and inter-assay precisions were within 3.88 % CV."

4. Line 195: It says "triple quadruple mass spectrometer ". Don't you mean "triple quadrupole"?
Yes, thank you for catching this error. This has been corrected in the manuscript.

5. Line 230: In this secΘon you listed a lot of specific results that are beΣer represented in tables. Do not repeat results in your results text if it is already listed in tables. Just refer to the tables and make some summary comments.
Text represented in Table 1 was removed from this section to avoid duplication of information.

6. Table 1: Do not say "plus or minus" one standard deviaΘon when lisΘng results in a table. This is not staΘsΘcally accurate. List the standard deviaΘon of your sample in parentheses next the mean value. Line 249: This secΘon addresses "Effect of Spironolactone Dosage". Are the results listed in table 1 dose proporΘonal? Do the metabolites measured increase by

approximately 2 fold, with the increase in dose? As it states in line 319, there is no apparent relaΘonship. Line 358 also acknowledges the lack of dose effect.
This correction has been made to table 1 in the manuscript and is reflected in the table heading and the body of the table. The observation regarding dose proportional effect of spironolactone on TMS and canrenone concentrations is insightful. Examining each of the two study periods separately, the fold-change in estimated canrenone exposure following administration of 4 mg/kg spironolactone compared to 2 mg/kg at T1 (immediately prior to spironolactone dosing) and T2 (5 hours post-spironolactone dose) was found to be 2.4 and 1.8 respectively, for D7; and 1 (T1) and 1.6 (T2) for D28. Concerning TMS, the fold-change in estimated exposure after 4 mg/kg spironolactone dosing in comparison to 2 mg/kg at T1 and T2 was measured at 2.4 and 2.4, respectively, for D0; and 1.1 (T1) and 1.6 (T2) for D28.

Pooling data from all study periods, the fold-change in estimated canrenone exposure following a doubling of the oral spironolactone dose stood at 1.4 and 1.7 at T1 and T2, respectively. For TMS, these estimates were 1.7 (T1) and 2.0 (T2). Taken together, these findings suggest a quasi-proportional relationship between the exposure of active spironolactone metabolites and the administered dose of spironolactone. Nonetheless, it is essential to approach the conclusions of this study with caution due to the study's inherent limitation, including a small subject pool and the utilization of a sparse sampling approach (limited to two timepoints in this instance).

Our data suggest that the effect of spironolactone active metabolites on biomarkers of the RAAS are not dose-proportional, with a plateauing of the effect already at 2 mg/kg/day of spironolactone.

7. The discussion secΘon is quite long. It is oŌen observed that when there is a lack of significance in a study, or if results do not agree with an author's assumpΘons, the discussion is quite long to explain why this may have occurred (unnecessary speculaΘon). However, your discussion can be shortened considerably by simply acknowledging that you do not know why these results occurred. Avoid unnecessary speculaΘon to shorten your discussion.
The authors acknowledge the length of the discussion section reflects the lack of significance in the study dataset. The discussion section was modified to remove any unnecessary speculation while also addressing reviewer #2 comment 3 requesting a more thorough explanation for the observed minimal effects of spironolactone on RAAS analytes in the study dataset. Specifically, the authors removed the statement "this may represent a true increase in adrenal responsiveness to AngII and subsequently increased aldosterone production secondary to feeding" from the manuscript's discussion of the effects of feeding on the RAAS.

8. Overall: I think the study is worthy of publicaΘon but there are many limitaΘons. The authors have cited these limitaΘons (small sample, normal healthy dogs, etc.). But overall, based on these results we cannot conclude that spironolactone has a clear benefit in dogs, parΘcularly on the RAAS cascade. It is unclear, based on this evidence, how administraΘon of spironolactone is

assumed to be beneficial in dogs with heart disease, and recommended, without quesΘon, in some protocols. Moreover, how did the products on the market get approved by regulatory authoriΘes? Perhaps these points deserve menΘon by the authors in their discussion.
The study reported in this manuscript was not designed to assess clinical benefit of spironolactone in dogs. The benefit to dogs with heart disease must be shown in dogs with disease and must evaluate clinical outcomes. This study was an initial exploratory study looking at short-term RAAS outcomes for increasing doses of spironolactone. These data can be used to optimize future clinical trials that look at clinical endpoints by showing that we do not need to use doses of spironolactone higher than 2mg/kg/day. Additionally, in the clinical patient spironolactone is typically not used as monotherapy, and its RAAS effect (e.g. preventing ABT) could be more profound in the context of concurrent ACE inhibition. The discussion section of the manuscript was modified to reflect this feedback (discussion paragraph 1; line 387 in "Revised Manuscript with Track Changes").

The regulatory approval of spironolactone for use in dogs was obtained prior to the availability of RAAS fingerprint analysis and therefore did not directly evaluate the effects of spironolactone on individual RAAS analytes (citation #2 and #16 in the study). At the time, the effect of different doses of spironolactone in healthy Beagle dogs on urinary sodium and potassium levels was used as a surrogate marker of degree of mineralocorticoid receptor antagonism for the registration and approval of spironolactone (citation #25 in the study). However, these surrogate markers may not be an accurate representation of the cardiovascular effects of the drug. This study is the first to provide data using the RAAS fingerprint in healthy dogs treated with spironolactone and provides a better understanding of the direct effects of spironolactone on the individual components of the RAAS than previous studies.

Reviewer #2
1. Authors mentioned several claims in introduction section regarding the association of spironolactone with reduced risk of cardiac morbidity and mortality in humans and dogs with CHF. However, there are no specific references provided for these statements. While the introduction introduces the concept of aldosterone breakthrough (ABT) in the context of ACE-I treatment, it fails to provide a comprehensive explanation of ABT and its underlying mechanisms. Furthermore, this section contains certain ambiguous statements which may require further clarification. For example, the mention of "genetic mutations in ACE" as a proposed mechanism of ABT lacks context and requires more elaboration.
The authors cite the following studies demonstrating reduced risk of cardiac morbidity and mortality in humans and dogs with CHF:
Citation #1: Pitt B, Zannad F, Remme WL, Cody R, Castaigne A, Perez A, et al. The effect of spironolactone on morbidity and mortality in patients with severe heart failure. New Engl J Med. 2008;341: 709–717.

> **This study was terminated early due to an interim analysis demonstrating that spironolactone significantly reduced the risk of death by 30% among human**

patients with severe heart failure and a left ventricular ejection fraction < 35%. Patients in the spironolactone group also had a significant improvement in their New York Heart Association functional class.

Citation #2: Bernay F, Bland JM, Ha J, Baduel L, Combes B, Lopex A, et al. Efficacy of spironolactone on survival in dogs with naturally occurring mitral regurgitation caused by myxomatous mitral valve disease. J Vet Intern Med 2010;24: 331–341.

This study demonstrated spironolactone treatment in dogs with myxomatous mitral valve disease significantly decreased the risk of reaching the composite endpoint (cardiac death, euthanasia because of mitral regurgitation, and worsening mitral regurgitation) by 55% (HR = 0.45). Spironolactone treatment reduced the risk of cardiac-related death or euthanasia in this study population by 69% (HR = 0.31).

Citation #3: Coffman M, Guillot E, Blondel T, Garelli-Paar C, Feng S, Heartsill S, et al. Clinical efficacy of benazepril and spironolactone combination in dogs with congestive heart failure due to myxomatous mitral valve disease: The Benazepril Spironolactone Study (BESST). J Vet Intern Med 2021; 1–15.

This study BESST demonstrated that treatment with combination spironolactone + benazepril in dogs significantly reduced risk of dying or worsening from cardiac causes by 27% (HR = 0.73) compared to benazepril treatment alone.

Citation #14: Laskary A, Fonfara S, Chambers H, O'Sullivan ML. Prospective clinical trial evaluating spironolactone in Doberman pinschers with congestive heart failure due to dilated cardiomyopathy. J Vet Cardiol 2022;40: 84–98.

This study demonstrated that the development of atrial fibrillation was significantly reduced in Doberman pinscher dogs with congestive heart failure secondary to dilated cardiomyopathy receiving spironolactone treatment when compared to those receiving placebo.

A more detailed description of aldosterone breakthrough has been added to strengthen the introduction section of the manuscript. Clarification regarding previously documented genetic mutations in ACE was also added to this section of the manuscript.

2. The study used a total of ten Beagle dogs, five in each dosing group, for the complete cross-over (AB/BA) two-arm design. While the authors mentioned random allocation to dosing groups, they did not elaborate on the method used for randomization or any power analysis to determine the sample size. It would be more appropriate to provide more details on the randomization process and justify the sample size to statistical design to draw meaningful conclusions.
The randomization was performed in R version 4.2.1 using the package "psych" and the function block.random. Clarification regarding the randomization process was added to the revised manuscript.

3. The authors mentioned that the effects of spironolactone treatment on circulating RAAS analytes in healthy dogs were minimal and varied between study periods and as a function of time and feeding status. However, the discussion does not provide a thorough explanation for the observed minimal effects. While the study provides valuable insights into spironolactone's effects on RAAS in healthy dogs, the discussion should also discuss future research directions and potential areas for further investigation.
The discussion section was modified to include a more thorough explanation for the observed minimal effects while being mindful of reviewer #1 comment 7 suggesting avoidance of any unnecessary speculation in the manuscript discussion.

4. Safety profile of the studied drugs i.e. spironolactone should be addressed.
The safety profile of spironolactone was expanded upon in the discussion section to include mention of previously documented adverse events associated with spironolactone treatment in humans. Availability of previously documented side effects of spironolactone in dogs are limited.

5. Limitation of the study should be discussed in limitation section of the manuscript.
Study limitations are addressed in the final paragraph of the discussion section (discussion paragraph 7; line 483 in "Revised Manuscript with Track Changes"). A separate study limitations section was not created in accordance with the style of other similar publications in PLOS ONE.

6. Authors should describe the Future perspective and clinical significance of the study.
The authors recommend future evaluation of circulating RAAS analytes in dogs with underlying RAAS activation, such as those with heart disease, following spironolactone treatment at broader dose ranges. These recommendations are detailed in the conclusion section of the manuscript.

7. Authors should add abbreviation list used in the manuscript.
The authors have added an abbreviation list to the start of the manuscript that includes all abbreviations referenced throughout the manuscript.
