# Supplementary material for: Comprehensive characterization of the effect of mineralocorticoid receptor antagonism with spironolactone on the renin-angiotensin-aldosterone system in healthy dogs
Source: PLoS One. 2024 Feb 23;19(2):e0298030. doi: 10.1371/journal.pone.0298030 (PMC10890738; doi:10.1371/journal.pone.0298030)
Supplement: Attachment — Submitted filename: Response to Reviewers.docx [file pone.0298030.s003.docx]

Manuscript: Comprehensive characterization of the effect of mineralocorticoid receptor antagonism with spironolactone on the renin-angiotensin-aldosterone system in healthy dogs

Response to PLOS ONE reviewers: PONE-D-23-07873R1

Dear PLOS ONE reviewers,

Thank you for the continued consideration of our manuscript “Comprehensive characterization of the effect of mineralocorticoid receptor antagonism with spironolactone on the renin-angiotensin-aldosterone system in healthy dogs.” We appreciate the time and effort that you put into reviewing our previous edits and continuing to provide insightful feedback. Please see our responses below, in bold, to the comments provided.

Reviewer #2:

I am pleased to recommend the acceptance of the manuscript for publication, as the authors have diligently addressed all the comments and concerns raised during the review process. The revisions made have significantly improved the quality and clarity of the article.

**Thank you for taking the time to provide constructive feedback that improved the quality and clarity of the article. We are sincerely grateful for your time and expertise.**

Reviewer #3:

The manuscript entitled “Comprehensive characterization of the effect of mineralocorticoid receptor antagonism with spironolactone on the renin-angiotensin-aldosterone system in healthy dogs” describes the effects of a mineralocorticoid receptor antagonist on the plasma levels of numerous RAAS components in dogs.

The research question addressed is highly relevant for veterinary medicine with increasing numbers of dogs being affected by heart disease and treated with mineralocorticoid receptor antagonists. The manuscript is well written and the interpretation of the results is scientifically sound. The low number of dogs enrolled as a major limitation of the study is already being discussed by the authors and is a common problem in this kind of studies. Hence, conducting this study with a homogenous group of n = 10 dogs and even enhancing the outcome by the elegant crossover design must be considered an achievement.

I have only minor comments and would recommend accepting the manuscript for publication.

Minor comments:
1) L.90-99: At least one reference for all the information on the RAAS should be added.

**The following citation was added to clarify and support the information on the RAAS in L90-99:**

**Citation #15: Hammond HK, Ames MK, Domenig O, Scansen BA, Tsang Yang N, Wilson MD, Sunshine E, Brunk K, Masters A. The classical and alternative circulating renin-angiotensin system in normal dogs and dogs with stage B1 and B2 myxomatous mitral valve disease. J Vet Intern Med 2023;37:875–886.**

2) L. 176: “red top tube” is not very specific, as this may vary.

**The language in L176 was changed to “additive-free tube” in order to be consistent with the language previously used in the methods section and to most accurately describe the tube type used.**

3) Although there is a statistically significant difference in some values for RAAS plasma levels, the biological relevance of an increase by factor 1.04 (and generally < 2) is highly questionable. This should be stated clearly in the discussion.

**A comment was added in the limitations section of the paper at L470 addressing the unknown relationship between statistical relevance and biological relevance in our data given the changes from baseline where typically < 2 fold.**

4) L.480: warrant (not warrants)

**This change was made in L480.**
